# Long-term PM_2.5_ exposure and sepsis mortality in a US medicare cohort

**DOI:** 10.1186/s12889-022-13628-5

**Published:** 2022-06-18

**Authors:** Trenton J. Honda, Fatemeh Kazemiparkouhi, Trenton D. Henry, Helen H. Suh

**Affiliations:** 1 School of Clinical and Rehabilitation Sciences, Northeastern University, 360 Huntington Avenue, Boston, MA 02115 USA; 2 Department of Civil and Environmental Engineering, Tufts University, Medford, MA USA; 3 Division of Public Health, Department of Family and Preventive Medicine, University of Utah, Salt Lake City, UT USA

**Keywords:** Sepsis, Air pollution, Chronic exposure, Particulate matter

## Abstract

**Background:**

Risk factors contributing to sepsis-related mortality include clinical conditions such as cardiovascular disease, chronic lung disease, and diabetes, all of which have also been shown to be associated with air pollution exposure. However, the impact of chronic exposure to air pollution on sepsis-related mortality has been little studied.

**Methods:**

In a cohort of 53 million Medicare beneficiaries (228,439 sepsis-related deaths) living across the conterminous United States between 2000 and 2008, we examined the association of long-term PM_2.5_ exposure and sepsis-related mortality. For each Medicare beneficiary (ages 65–120), we estimated the 12-month moving average PM_2.5_ concentration for the 12 month before death, for their ZIP code of residence using well validated GIS-based spatio-temporal models. Deaths were categorized as sepsis-related if they have ICD-10 codes for bacterial or other sepsis. We used Cox proportional hazard models to assess the association of long-term PM_2.5_ exposure on sepsis-related mortality. Models included strata for age, sex, race, and ZIP code and controlled for neighborhood socio-economic status (SES). We also evaluated confounding through adjustment of neighborhood behavioral covariates.

**Results:**

A 10 μg/m^3^ increase in 12-month moving average PM_2.5_ was associated with a 9.1% increased risk of sepsis mortality (95% CI: 3.6–14.9) in models adjusted for age, sex, race, ZIP code, and SES. HRs for PM_2.5_ were higher and statistically significant for older (> 75), Black, and urban beneficiaries. In stratified analyses, null associations were found for younger beneficiaries (65–75), beneficiaries who lived in non-urban ZIP codes, and those residing in low-SES urban ZIP codes.

**Conclusions:**

Long-term PM_2.5_ exposure is associated with elevated risks of sepsis-related mortality.

## Introduction

Air pollution is an ubiquitous environmental exposure that has been consistently associated with adverse health outcomes and mortality in numerous studies, including lower respiratory infections [1, 2], and diabetes mellitus [3, 4]. However, there is a dearth of prior literature examining the impact of air pollution on high mortality risk medical conditions closely linked with these established health outcomes, such as sepsis [5]. Sepsis is an overwhelming and potentially life-threatening inflammatory response to microbial invasion, frequently bacterial, into normally sterile regions of the body. This extreme, dysregulated response is characterized by life-threatening organ dysfunction and is associated with a high risk of mortality [6]. Importantly, while sepsis is an acute event, the risk of sepsis increases appreciably in individuals with certain medical conditions that have been previously linked with air pollution exposure, including: Chronic lung disease, cardiovascular disease, cerebrovascular disease, diabetes, and hypertension [5]. Furthermore, recent models of disease trajectory demonstrate that sepsis mortality is specifically associated with many of these antecedent comorbidities, indicating that many known health effects of air pollution—namely diabetes and cardiovascular disease—might increase the risk of both developing and dying from sepsis [7].

A number of potential biological pathways through which these pollutants may impact the development and severity of sepsis have been described. For example, air pollution impacts the clearance of bacteria from lung tissue, suppresses aspects of the innate immune response in the lungs, and increases susceptibility to pulmonary infection from certain pathogens [8–10]. Importantly, sepsis from pulmonary origins is known to confer a significantly increased mortality risk relative to other origins. For example, one study found that sepsis originating from pneumonia was associated with a 76% increased risk of mortality (95% CI: 11%, 178%) relative to sepsis from other origins [11]. It is thus possible that the impact of air pollutants on respiratory infections directly impacts the risk of sepsis mortality. Indirectly, air pollution exposure might also increase the risk of sepsis mortality through its well-described effects on systemic inflammation and oxidative stress, resulting in perturbed immune responses that detrimentally impact the ability to clear the underlying infection [12].

Despite these established potential mechanisms, little is known about whether, and to what extent, air pollution may contribute to sepsis-associated mortality. Groves et al. (2020) found null associations between short-term air pollution exposures and sepsis-related ICU admissions in an Australian and New Zealand cohort (13). Likewise, Sarmiento et al. (2018) found null associations between short-term and long-term air pollution exposures and sepsis incidence in a US cohort [14]. However, the study design (case–control), selection criteria for controls, and the small size of the study (*n* = 1386 cases, *n* = 5544 controls) may have impacted their results. Likewise, in a US study (*n* = 444,928) examining the impact of ambient air pollution exposures on the risk of mortality among patients with sepsis, Rush et al. (2018) found no significant association between PM2.5 and mortality [15]. However, this study may be limited by exposure misclassification, as air pollution estimates were made at the county-level of the admitting hospital, and not the residence of the individual [15].

Given the tremendous morbidity and mortality burden linked to sepsis, identifying novel and modifiable risk factors is of utmost importance. The Centers for Disease Control and Prevention (CDC) reports that 1.7 million U.S. adults contract sepsis each year, resulting in 270,000 deaths annually [16]. Worldwide, it is estimated that over 30 million sepsis cases occur annually, resulting in 5.3 million deaths [17]. Sepsis is particularly harmful to vulnerable populations, such as the elderly, and reportedly incurs $22.4 billion in healthcare costs among American Medicare beneficiaries alone [13, 18].

To address the limitations in our understanding of the impact of air pollution on sepsis mortality, our study aims to examine the association of sepsis-related mortality and chronic exposure to PM2.5 within a cohort of 53 million U.S. Medicare beneficiaries between 2000 and 2008.

## Materials and methods

All methods were carried out in accordance with relevant guidelines and regulations, and only de-identified data were used. This study was approved by the Institutional Review Boards of Tufts University.

### Air pollution exposures

We estimated 12–60 month moving average PM2.5 concentrations using well validated GIS-based (Geographical Information System) spatio-temporal models that estimated daily PM2.5 exposures on a 6 km grid covering the conterminous US [19]. Model inputs included PM2.5 data from the U.S. Environmental Protection Agency (EPA), meteorological and geospatial covariates, and traffic-related PM2.5 estimates using a Gaussian line-source dispersion model [20]. The daily PM2.5 model performed well, with a cross-validation R2 of 0.76, with low bias and high precision.

To estimate non-traffic PM2.5, we used NO2 exposure estimates from land use regression models developed by Bechle et al. (2015) that estimated monthly NO2 exposure for census blocks with a high degree of accuracy and precision [21]. We then estimated PM2.5 from non-traffic sources using a two-stage approach, following the methods described in Wang et al. [22]. In the first stage, we linearly regressed 12-month moving average PM2.5 on 12-month moving average NO2 to estimate the amount of PM2.5 originating from non-traffic sources. In the second stage, we used the residuals from this regression as the exposure measure in Cox proportional hazard models.

For total PM2.5 and non-traffic PM2.5 we matched beneficiaries to the grid point closest to the ZIP code centroid most proximal to their residential address, and adjusted the assigned grid point to correspond to their current residence in the event of a reported change of address. As our main exposure window of interest, we assessed the impact of 12-month moving average exposure for both pollutants of interest. While all participants had valid PM2.5 measures assigned to their ZIP code of residence, NO2 estimates were available only for 91.2% of the Medicare population. As such, in our non-traffic PM2.5 models, we employed complete-case analyses.

### Mortality data

We compiled enrollment data from the Centers for Medicare and Medicaid Services for 53 million Medicare beneficiaries (ages 65–120) living in the conterminous US between 2000 and 2008. For each enrollee, we obtained beneficiary-specific information on date of birth, sex, race, ZIP code of residence, and survival. Using the International Classification of Disease (ICD-10) codes from the National Death Index, we extracted mortality from Streptococcal sepsis (A40) and other sepsis (A41).

### Covariates

Covariates were selected based upon their prior associations with sepsis mortality or air pollution. Individual level covariates included age, sex, and race/ethnicity. We categorized age into 1-year intervals, with 90 + years included as one age interval to avoid excessive zero counts. Sex was reported as a binary variable, and race/ethnicity was divided into the following categories based upon self-report: Asian, Black, Hispanic, and White. Area-level covariates included ZIP code and state-level SES, which were assessed using the annual mean gross adjusted income from the US Internal Revenue Service (IRS) Statistics of Income Division database [23]. Urbanicity (urban vs. non-urban) was assessed using Categorization B from the Rural Health Research Center (RHRC) [24].

For a subset of our Medicare population, we linked measures from Selected Metropolitan/Micropolitan Area Risk Trends of the BRFSS (Behavioral Risk Factor Surveillance System), which provide data on health-related risk behaviors for 378 US counties. In our analysis, 28.4 million beneficiaries lived in ZIP codes (13,893 of 38,715) located in a county with BRFSS data. Covariates available for this sub-population included monthly county-level prevalence of current smokers, non-whites, diabetics, heavy drinkers (i.e., > two drinks per day), asthma and mean body mass index.

### Statistical analysis

We examined the associations between 12-month moving average PM2.5, non-traffic PM2.5, and sepsis-related mortality using Cox proportional hazards (Cox PH) models with strata for age, sex, race (white/non-white) and ZIP code, controlling for ZIP code and state SES (Eq. 1).

1 $$h\left({t}_{i}|{X}_{i}, {s}_{i}\right)={{h}_{0}}_{s}\mathrm{exp}\left({{\varvec{\beta}}}^{T}{{\varvec{x}}}_{i}\right)$$

where $i$ represents each individual, ${{h}_{0}}_{s}$ is a stratum-specific baseline hazard function, ${y}_{i} = min({t}_{i}, {c}_{i}),$ where ${t}_{i}$ is event time and ${c}_{i}$ the right-censoring time for each individual $i$, and ${{\varvec{x}}}_{i}={\left({x}_{i1}, {x}_{i2}, \dots , {x}_{ip}\right)}^{T}$ represents a vector of covariates for the individual $i$, and ${\varvec{\beta}}={\left({\beta }_{1}, {\beta }_{2}, \dots , {\beta }_{p}\right)}^{T}$ is the vector of estimated model parameters [22].

Our implementation of Cox PH for large-scale data had been described in detail previously [22] and hosted on GitHub (https://github.com/Rainicy/survival). We examined effect modification using interaction terms for variables previously shown to be associated with air pollution exposure, sepsis mortality, or both, including: age, sex, race, urbanicity and urban ZIP code SES categories. All results are expressed as the hazard ratio (HR) per 10 μg/m3 increase in 12-month average PM2.5 and non-traffic PM2.5.

In sensitivity analyses, we fit air pollution-sepsis mortality models that additionally adjusted for behavioral risk factors from the BRFSS for the subset of beneficiaries for which such data were available. We specifically examined potential confounding by monthly county-level prevalence of current smokers, non-white race, diabetics, heavy drinkers (i.e., > two drinks per day), asthma and mean body mass index. Additionally, we examined whether PM2.5-associated HRs varied with the length of the exposure window, examining the association of PM2.5 exposures based upon 24, 36, 48, and 60-month moving averages. Missing data were addressed using complete case analyses; all statistical analyses were conducted using Java 8.

## Results

Our study population includes approximately 53 million Medicare enrollees living in nearly 39,000 US ZIP codes between 2000–2008 (Table 1). During the study period, more than 228,000 sepsis-related deaths were reported. The overall mean 12-month PM2.5 concentration was 10.32 μg/m3 (SD = 3.15).

**Table 1:** Baseline demographics for Medicare beneficiaries and death time demographics for Sepsis-related death, US 2000—2008

	**Enrollee**	**Death due to Sepsis**
**Persons,** n (%)	52,902,921 (100.0)	228,439 (100.0)
**ZIP code,** n (%)	38,715 (100.0)	24,934 (62.6)
**Age,** n (%)		
< = 75	38,534,953 (72.8)	60,449 (26.5)
> 75	14,367,968 (27.2)	167,990 (73.5)
**Sex,** n (%)		
Female	29,928,520 (56.6)	131,984 (57.8)
Male	22,974,401 (43.4)	96,455 (42.2)
**Race,** n (%)		
Asian	844,228 (1.6)	1,613 (0.7)
Black	4,523,321 (8.6)	34,535 (15.1)
Hispanic	958,465 (1.8)	3,688 (1.6)
White	45,495,610 (86.0)	185,714 (81.3)
Other	1,081,297 (2.0)	2,889 (1.3)
**Urbanicity**^a^**,** n (%)		
Urban	39,656,002 (75.0)	173,514 (76.0)
Nonurban	11,897,208 (22.5)	48,597 (21.3)
**Race (Urban), n (%)**		
Asian	811,611 (1.5)	1,536 (0.7)
Black	3,725,768 (7.0)	28,015 (12.3)
Hispanic	841,382 (1.6)	3,161 (1.4)
White	33,401,957 (63.1)	138,782 (60.8)
Other	875,284 (1.6)	2,020 (0.9)
**Income (Urban), n(%)**		
Low	7,818,031 (14.8)	60,660 (26.6)
Middle	15,468,091 (29.2)	56,429 (24.7)
High	16,369,880 (30.9)	56,425 (24.7)
**Region**		
**Northeast**	10,494,342 (19.8)	61,462 (26.9)
**South**	12,485,446 (23.6)	96,040 (42.0)
**Midwest**	19,053,623 (36.0)	50,780 (22.2)
**West**	10,869,510 (20.5)	20,157 (8.8)
**With NO**_**2**_^b^ **Data,** n (%)	48,224,895 (91.2)	192,849 (84.4)
**With BRFSS**^c^ **Data,** n (%)	28,416,054 (53.7)	81,004 (35.5)

*Abbreviations*: *NO*_*2*_ Nitrogen dioxide, *BRFSS* Behavioral Risk Factor Surveillance System

^a^Urbanicity data was available for 29,572 ZIP codes covering 97.5% of population

^b^NO_2_ data first become available in 2001

^c^BRFSS data first become available in 2002

**Fig. 1:**
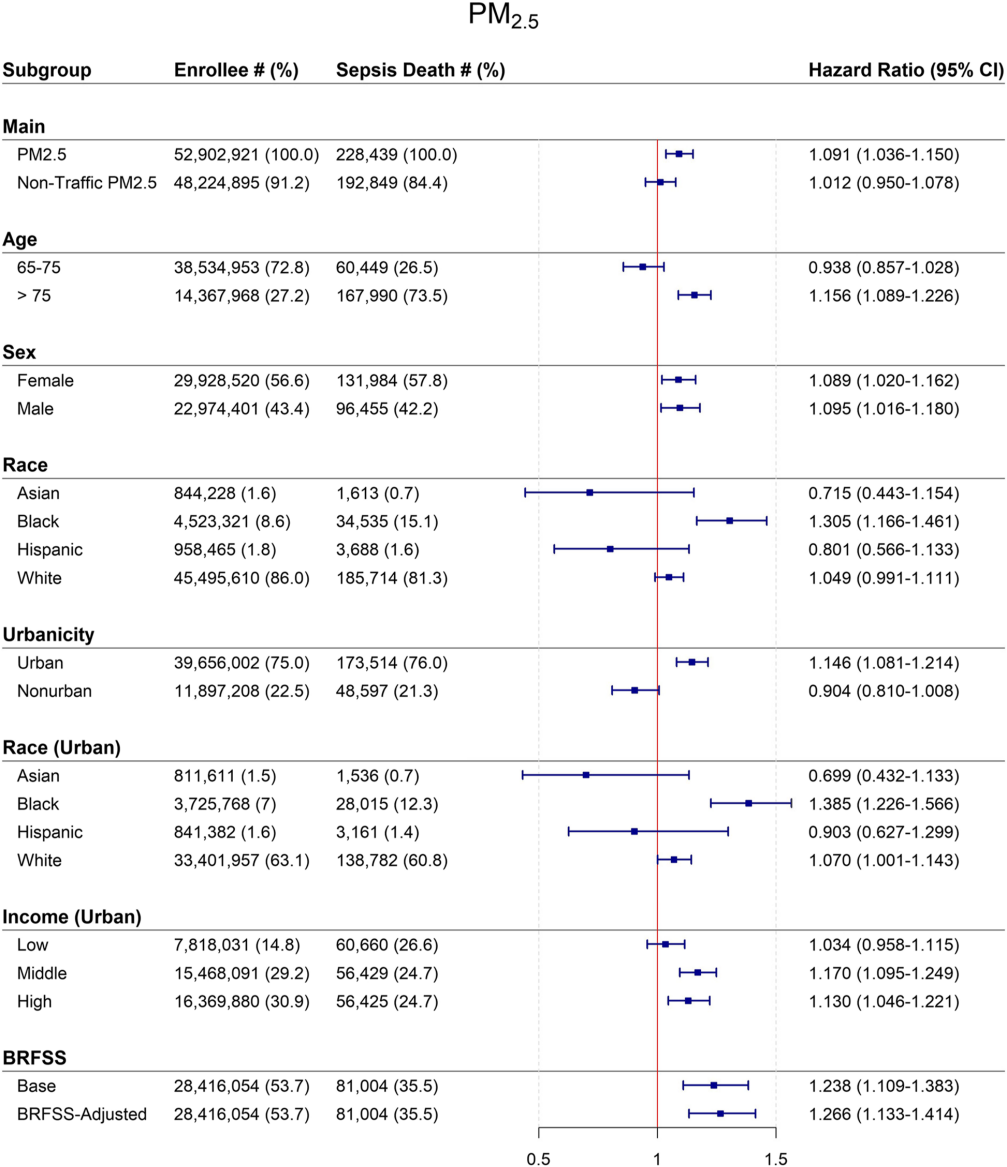
Mortality hazard ratios* (95% CI) associated with a 10 μg/m3 increase in 12-month average PM2.5 and non-traffic PM2.5† for entire population and by subgroup, US 2000—2008. Abbreviations: *CI* Confidence interval, *PM*_*2.5*_ Particles with aerodynamic diameters < 2.5 μm, *BRFSS* Behavioral Risk Factor Surveillance System. ^*^ Estimated using Cox PH models with strata for age (1 year age categories with 90 + year old as one category), sex (male, female), race (white, non-white) and ZIP Code (38,715 ZIP codes), adjusted for ZIP code and state SES. ^†^ While all participants had valid PM_2.5_ measures assigned to their ZIP code of residence, NO_2_ estimates were available only for 91.2% of the Medicare population

Figure 1 shows HRs associated with 12-month PM2.5 and non-traffic related PM2.5 for sepsis-related mortality for the entire population and by subgroup. In fully adjusted models, a 10 $\mu g$/m3 increase in exposure to PM2.5 increased risk of dying from sepsis by 9.1% (HR:1.091, 95% CI: 1.036–1.150). In comparison, HRs for non-traffic PM2.5 and sepsis mortality, while positive, were statistically insignificant (HR 1.012, 95% CI: 0.950–1.078).

We found risks of death to vary by beneficiary characteristics. Race had the greatest impact on sepsis-related mortality risks. HRs were highest for Black beneficiaries (HR 1.305. 95% CI: 1.166–1.461), while associations for White, Hispanic and Asian participants were not statistically significant. However, when restricting the race analysis to urban ZIP codes, we found stronger associations for both Black (HR 1.385, 95% CI: 1.226–1.566) and White (HR 1.070, 95% CI: 1.001, 1.143) participants, with the effect estimates for White participants positive and statistically significant. Associations were null for all racial groups when restricted to non-urban ZIP codes. By age, HRs were higher for participants > 75 years (HR 1.156, 95% CI: 1.089–1.226) as compared to younger (65–75) beneficiaries (HR 0.938, 95% CI: 0.857–1.028). Differences in risks of mortality by sex were small, with significant positive risks for both men and women.

We additionally found beneficiaries living in urban as compared to non-urban ZIP codes to have higher mortality risks, with PM2.5-associated risk null for beneficiaries living in non-urban areas. Likewise, PM2.5-associated risks were highest for beneficiaries living in high and middle income urban neighborhoods, and null in low income urban neighborhoods.

## Sensitivity analyses

In sensitivity analyses, we explored whether our effect estimates were robust to controlling for potential health behavior confounders, and longer-term exposure windows. Potential confounding by health behaviors was estimated with and without adjustment of BRFSS covariates using the smaller population subset. We observed that PM2.5 associated HRs were minimally and only nominally different in models adjusting for BRFSS covariates as compared to those from our main models (1.266 versus 1.238). Additionally, we examined PM2.5-sepsis mortality associations for longer exposure windows of 24–60 months, for which each remained statistically significant, with the largest magnitude association observed for the 60-month moving average exposures (Table 2).

**Table 2:** Mortality hazard ratios^a^ (95% CI) associated with a 10 μg/m^3^ increase in 12- to 60-month moving average PM_2.5_^b^, US 2005—2008

Exposure Window	Hazard Ratio (95% CI)
12-month	1.071 (1.016–1.130)
24-month	1.124 (1.054–1.198)
36-month	1.196 (1.116–1.283)
48-month	1.204 (1.116–1.300)
60-month	1.210 (1.114–1.315)

*Abbreviations*: *CI* Confidence interval, *PM*_*2.5*_ Particles with aerodynamic diameters < 2.5 μm

^a^Estimated using Cox PH models with strata for age (1 year age categories with 90 + year old as one category), sex (male, female), race (white, non-white) and ZIP Code (38,715 ZIP codes), adjusted for ZIP code and state SES

^b^Subset of ZIP codes with complete data of 12- to 60- month moving average

## Discussion

We assessed the impacts of long-term particulate air pollution exposure on sepsis-related mortality in the largest cohort examined to date, evaluating almost 53 million Medicare beneficiaries and more than 228,000 deaths in nearly 39,000 ZIP codes across the US. By virtue of its large size, we were able to examine PM2.5-associated impacts on sepsis mortality for which current evidence is sparse. We showed that a 10-μg/m3 increase in 12-month moving average PM2.5 exposure was associated with a 9.1% increased risks of sepsis mortality in age, sex, race, ZIP code, and SES-adjusted models. The magnitude of the PM2.5-associated HRs increased as exposure windows increased from 12- to 60-months, and were observed to be highest in Black beneficiaries. All associations were robust when adjusted for BRFSS covariates in sensitivity analyses. Risks associated with non-traffic PM2.5 were lower as compared to that for our total PM2.5 models and associations were non-statistically significant, suggesting that PM2.5 specifically related to combustion sources are responsible for the observed adverse effects on sepsis mortality.

To our knowledge, this is the first study to report positive and statistically significant associations of PM2.5 exposure and sepsis-related mortality in an American population. Previously, Rush et al. (2018) used data from the 2011 Nationwide Inpatient Sample (NIS) cohort to examine the impact of chronic O3 and PM2.5 exposure on mortality in a cohort of patients all of whom had sepsis at baseline [15]. They reported a null association for PM2.5 sepsis-related mortality, in contrast to our study, but found significant and elevated O3-associated risks (1.04; CI: 1.03–1.05). A number of important differences between Rush et al. (2018) and our study may account for our different findings. While our cohort was limited to Medicare beneficiaries (65 years and older), Rush et al., included a far wider age range, including patients 18 years and older, suggesting that PM2.5-associated risks are most relevant to older adults, as evidenced by our effect modification results by age. Second, Rush et al. assigned air pollution exposures based upon the hospital ZIP code, which may result in greater exposure error as compared to our study, which is based on the participants’ residential ZIP codes, accounting for residential moves over time. Third, their study participants were limited only to people with sepsis, exploring whether ambient pollutant exposures were associated with mortality among people with sepsis. As a result, their study examined the role of short-, rather than as in our study, long-term, PM2.5 exposures on sepsis mortality risks. Finally, while Rush et al. included patients living in 28 states, our study includes all 48 states of the contiguous US and Washington DC which affords a much larger and more representative sample.

A number of additional studies have investigated associations between short-term air pollutants and sepsis incidence and hospital admissions due to sepsis with mixed results. Sarmiento et al., (2018) found null associations between short (30-day) and long (1-year) term air pollution exposures and incidence of community acquired sepsis in a small, US case–control study (1386 cases, 5,544 age and sex-matched controls) [14]. Null findings in their study may result from their smaller sample size and reliance on self-reporting of hospitalization. Similarly, in an Australian cohort, Groves et al. (2020) investigated hospital admissions due to sepsis associated with PM2.5 (RR: 0.910, 95% CI: 0.822, 1.008), PM10 (RR: 0.982, 95% CI: 0.954, 1.011) and NO2 (RR: 0.984, 95% CI: 0.895, 1.081) exposure and found null results [13]. It is possible that the relatively small sample number of cases (10,725) and substantially different population and health care systems might contribute to the different findings. This is supported by the findings of Wei et al. (2019) who reported significant, positive associations between short-term PM2.5 and hospital admissions for septicemia in a large (*n* = 95,277,169) claims-data analysis of US Medicare data, in which the sample size and population are more similar to our cohort [25].

We found significant effect modification by age, urbanicity, SES, and race. For age, we found adults over 75 years to be at greater risk as compared to participants between 65 and 75 years. Increased risk for the oldest beneficiaries may reflect an exaggerated response to air pollution-associated alterations in lung function, age-related immune senescence, or both [26, 27]. Participants who live in urban ZIP codes also experienced higher mortality rates than those living in less urban or rural ZIP codes. This may be explained by differences in PM composition in rural versus urban environments, with combustion-related PM2.5 (1) comprising a smaller fraction of PM2.5 in rural as compared to urban environment and (2) more closely associated with sepsis-mortality risks. However, among these urban beneficiaries, the PM2.5-associated sepsis mortality risks were higher for individuals living in high and middle as compared to low SES neighborhoods. Precise reasons for this are unclear, however we are not the first paper to find such an association [28]. Possible explanations include differential access to care, as well as higher levels of competing risks of mortality among lower SES groups. As sepsis is treated in hospital settings, and sepsis mortality recorded in our data likely therefore occurs in hospitals, it is possible that this reflects differential access to higher levels of medical care for those in middle and high versus low-income neighborhoods, or competing risks due to lower life expectancy in lower income neighborhoods, potentially related to a higher burden of antecedent comorbidities [29]. It is also possible that our use of ZIP code level SES results in residual confounding, as prior studies have found differential mortality effects for air pollutants when examining the impact of coarser versus finer geographic resolutions of SES measures [30].

We also found that associations between PM2.5 and sepsis mortality differed by race. Hazard ratios in Black participants were 6.2 times higher as compared to White participants, which is consistent with prior literature examining disparities in sepsis mortality by race [31–35]. Importantly, prior research has also found that chronic, comorbid conditions that alter immune function—and thus confer increased sepsis risk—are also more prevalent among non-White sepsis patients [35]. Additionally, a growing literature demonstrates that Black populations experience higher cause-specific mortality risk from air pollution exposures relative to other racial/ethnic groups [22, 36, 37]. These increased risks among Black populations likely reflect the significant and detrimental impacts of current and historical systemic racism, and the resultant differential access to healthcare and differential exposure to environmental toxicants [35, 38].

The positive associations we observed are consistent with biological pathways through which PM2.5 is known to influence health. A number of laboratory studies have also found that air pollution impacts the clearance of bacteria from lung tissue, suppresses aspects of the innate immune response, and increases susceptibility to pulmonary infection from certain pathogens [8–10]. As sepsis most often originates from pulmonary transmigration of bacteria into the blood, the impact of air pollution on the clearance of, and susceptibility to, pulmonary pathogens may directly impact sepsis risk. Indeed, a recent study found that sepsis from pulmonary origins was associated with 76% increased risk of mortality (95% CI: 11%, 178%), relative to sepsis from non-pumonary sources [11], suggesting that that air-pollution associated perturbations of pulmonary microbial clearance and immune defences may be particularly life-threatening.

Beyond its direct pulmonary effects, air pollution exposure is also known to result in systemic inflammation and oxidative stress [12], with a number of previous studies showing perturbed systemic cytokine production associated with elevated exposure [39, 40]. The chronic, systemic inflammation that results from long-term air pollution exposure is a known risk factor for many intermediate clinical conditions that alter immune responses and predispose individuals to sepsis [5], including: cardiovascular disease [41], cerebrovascular disease [42], hypertension [43, 44], and diabetes [3]. Given the central role of systemic inflammation, cytokine production, and perturbed immune function in sepsis, it is plausible that air pollution impacts the development of both sepsis and sepsis risk factors, ultimately increasing the risk of sepsis mortality by interacting with or potentiating these underlying pathophysiologic processes [45, 46].

Our study had several limitations. First, while we used ambient PM2.5 estimates based upon well validated models, we expected non-differential exposure misclassification which might dilute effect sizes in the present study [19]. Second, our study examined only adults > 65 years living in the U.S., limiting generalizability to younger or non-U.S. populations. Third, while we had information on individual level age, sex, and race/ethnicity, we were unable to adjust for potential individual level SES or behavioral confounders. Fourth, our findings may not be immediately comparable to other sepsis mortality studies because the case definition for sepsis can vary widely [6, 18, 47, 48] and be confounded with other organ-system injuries [49–52]. These limitations are counterbalanced by a number of important strengths. Our cohort’s large size and use of exposure models to estimate PM2.5 exposures for each ZIP code allowed for sufficient statistical power to estimate associations in understudied racial/ethnic populations.

## Conclusions

We found positive and statistically significant associations of PM2.5 exposures and sepsis-related mortality in a large cohort of older, US adults. Associations were strongest among Black participants, older adults, and those living in urban areas. Our findings suggest that air pollution may be an important, understudied contributor to sepsis-related mortality in the US.

## Data Availability

The data that support the findings of this study are available from [third party name] but restrictions apply to the availability of these data, which were used under license for the current study, and so are not publicly available. Data are however available from the authors upon reasonable request and with permission of [third party name].
